# Lack of Physiological Depth Patterns in Conspecifics of Endemic Antarctic Brown Algae: A Trade-Off between UV Stress Tolerance and Shade Adaptation?

**DOI:** 10.1371/journal.pone.0134440

**Published:** 2015-08-07

**Authors:** Iván Gómez, Pirjo Huovinen

**Affiliations:** Instituto de Ciencias Marinas y Limnológicas, Facultad de Ciencias, Universidad Austral de Chile, Valdivia, Chile; Mount Allison University, CANADA

## Abstract

A striking characteristic of endemic Antarctic brown algae is their broad vertical distribution. This feature is largely determined by the shade adaptation in order to cope with the seasonal variation in light availability. However, during spring-summer months, when light penetrates deep in the water column these organisms have to withstand high levels of solar radiation, including UV. In the present study we examine the light use characteristics in parallel to a potential for UV tolerance (measured as content of phenolic compounds, antioxidant activity and maximum quantum yield of fluorescence) in conspecific populations of four Antarctic brown algae (*Ascoseira mirabilis*, *Desmarestia menziesii*, *D*. *anceps* and *Himantothallus grandifolius)* distributed over a depth gradient between 5 and 30 m. The main results indicated that a) photosynthetic efficiency was uniform along the depth gradient in all the studied species, and b) short-term (6 h) exposure to UV radiation revealed a high tolerance measured as chlorophyll fluorescence, phlorotannin content and antioxidant capacity. Multivariate analysis of similarity indicated that light requirements for photosynthesis, soluble phlorotannins and antioxidant capacity are the variables determining the responses along the depth gradient in all the studied species. The suite of physiological responses of algae with a shallower distribution (*A*. *mirabilis* and *D*. *menziesii*) differed from those with deeper vertical range (*D*. *anceps* and *H*. *grandifolius*). These patterns are consistent with the underwater light penetration that defines two zones: 0–15 m, with influence of UV radiation (1% of UV-B and UV-A at 9 m and 15 m respectively) and a zone below 15 m marked by PAR incidence (1% up to 30 m). These results support the prediction that algae show a UV stress tolerance capacity along a broad depth range according to their marked shade adaptation. The high contents of phlorotannins and antioxidant potential appear to be strongly responsible for the lack of clear depth patterns in light demand characteristics and UV tolerance.

## Introduction

Seaweed communities from the Antarctic Peninsula and adjacent islands are characterized by an exuberant abundance whose standing crops support most of the biogeochemical processes at coastal ecosystems [1–4]. The harsh Antarctic conditions restrict the distribution of the seaweeds mainly to the sublittoral zone between 0 and 50 m, where they have relatively constant physicochemical conditions to grow and photosynthesize [5]. A striking feature of various macroalgal species, especially of endemic brown algae, is their broad bathymetric range. In fact, species of the order Desmarestiales such as *Himantothallus grandifolius*, *Desmarestia anceps* and *D*. *menziesii*, as well as some fucoid species such as *Ascoseira mirabilis* and *Cystosphaera jacquinotii* can extend between 2 and 40 m [6–9]. This remarkable ability underlies efficient photobiological adaptations, especially very low light requirements for photosynthesis and growth of both early stages and adult thalli [10–12]. For example, minimum light demands for saturation and compensation of photosynthesis can be as low as 2 μmol m^-2^ s^-1^, while minimum irradiances for saturation of photosynthesis can be close to 10 μmol m^-2^ s^-1^ [11]. Based on photosynthesis versus light curves, it has been demonstrated that Antarctic seaweeds from shallow waters show higher saturated net photosynthesis (P_max_) and light requirements for photosynthesis (E_k_) than species from deeper zones, however, the photosynthetic efficiency (the initial slope of the P-I curve) is similarly high over an extended depth gradient [9,13]. The capacity of Antarctic algae to maintain a low respiration rate and a positive carbon balance at compensation irradiances tuned to the seasonal light conditions have been claimed as the factors explaining this shade adaptation [10,11,14,15].

Considering that Antarctic marine ecosystems are subjected to extreme variability in the physical environment, the vertical zonation of seaweeds clearly cannot be explained only by the light use capacity as other abiotic and biotic factors such as substrate characteristics, strong perturbation due to salinity, temperature, solar UV radiation, ice scouring and grazing, which act mostly in interaction, are also involved [12, 16–18]. Recent studies revisiting Antarctic seaweed zonation have added the potential for stress tolerance (e.g. to temperature, UV radiation, etc.) as a relevant physiological trait underlying this broad vertical distribution [9,19,20]. For example, algae from shallow water (0 and 5 m depth) showed up to 15% reduction in photosynthesis and 75% increase in DNA damage when exposed for 2 h to summer levels of UV radiation. In contrast, algae from deeper locations (30–40 m) exhibited up to 35% reduction in photosynthesis and DNA damage increased by up to 150%. However, in both groups of algae, a recovery from photodamage occurred, which was also correlated with the synthesis of photoprotective substances and antioxidant activity [9]. On the other hand, the activity of some antioxidant enzymes (e.g. SOD) of *Desmarestia anceps* was higher in conspecifics collected at shallower locations (5.5 m) compared to 13.5 m [19]. These results point to regulative physiological strategies in mitigating photodamage of sensitive molecules and processes, even in shade adapted species.

Global warming driven processes affecting coastal waters (e.g. ice melting and sediment run-off) will alter the incidence of solar radiation to the marine biota in ways that are not fully understood. Recent studies suggest that seaweeds have a capacity to occupy new ice-free substrates as a consequence of glacier retreat [21], but this colonization can be constrained by enhanced water turbidity and lower light penetration [22]. These results emphasize the need for more research focused on the bio-optical and photosynthetic characteristics in order to better estimate the impact of changes in the underwater light climate in this region, a basic requisite to define the range of depths where distinct populations of key seaweed species are effectively at risk or where they will find refuge from environmental stress.

The present study examines the physiological performance of four endemic Antarctic species (*Ascoseira mirabilis*, *Desmarestia menziesii*, *Desmarestia anceps* and *Himantothallus grandifolius*) with broad vertical distribution in the context of solar UV radiation. Given that biochemical and physiological mechanisms developed to cope with high solar radiation are superimposed onto the photobiological adaptations to low light (a trade-off operating along the entire depth gradient), we hypothesize that algae maintain a high potential for UV stress tolerance irrespective of their depth location. Using uni- and multivariate analysis we tested this prediction in conspecifics collected at three different depths in a range between 5 and 30 m. Light use characteristics (P-I parameters) related with shade adaptation and physiological responses related with UV stress tolerance (contents of phlorotannins, antioxidant activity and maximum quantum yield) were studied.

## Materials and Methods

### Sampling and site characteristics

**Fig 1:**
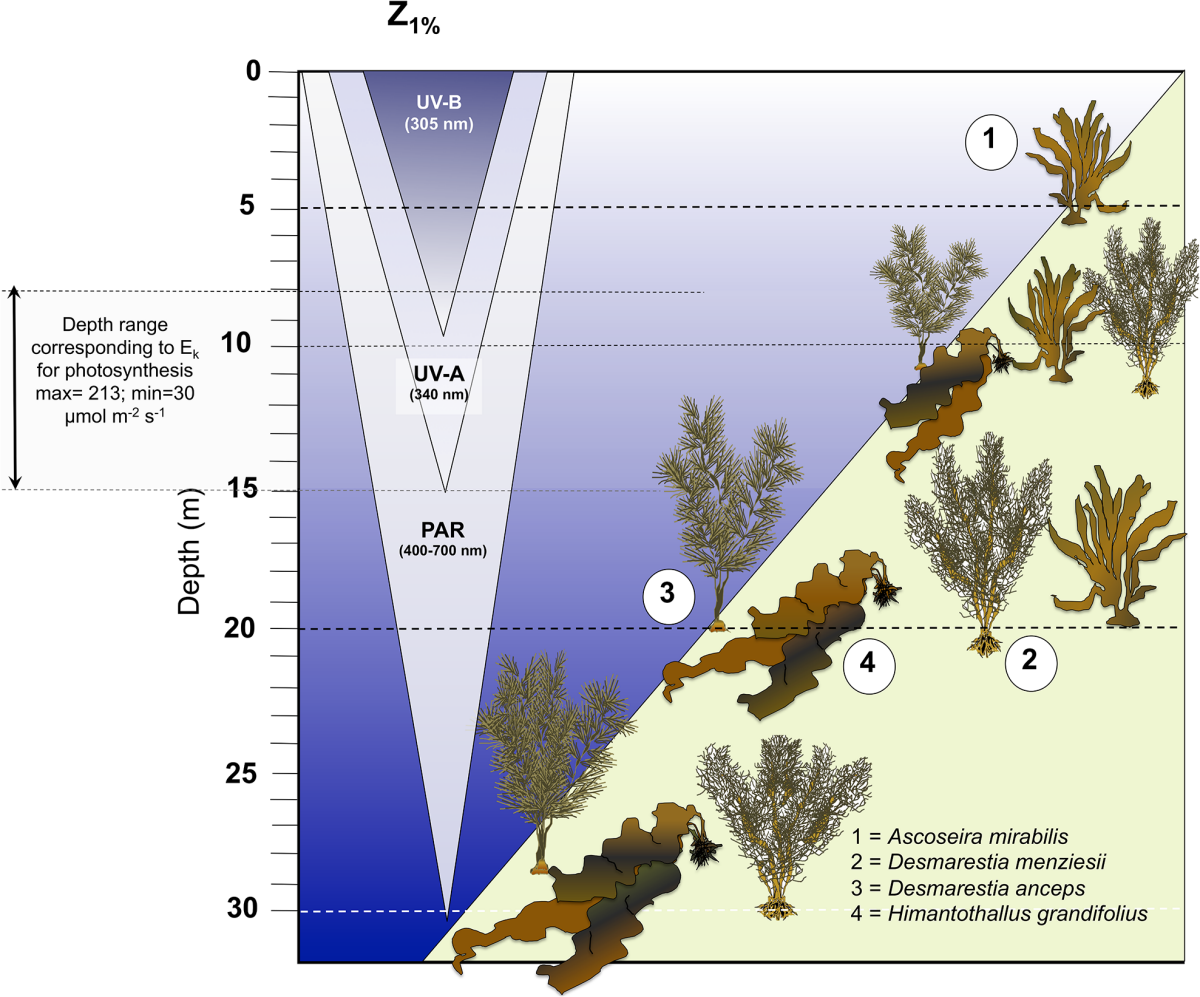
Scheme of the vertical distribution of the studied Antarctic macroalgae coexisting at King George Island indicating the summer light field scenario of relevant solar radiation wavelengths and depth ranges for saturation of photosynthesis (E_k_).

Field activities were granted by the Instituto Antártico Chileno (INACH) in accordance with the Protocol on Environmental Protection to the Antarctic Treaty. Non-destructive sampling was located outside of the Antarctic Specially Protected Areas (ASPAs) and no endangered or protected species were collected. Fronds of 6–9 individuals of *Ascoseira mirabilis*, *Desmarestia menziesii*, *Desmarestia anceps* and *Himantothallus grandifolius* were collected by Scuba diving during the austral summer (February 2013) from Fildes Bay, King George Island (62°12'S) from 5 to 30 m depths. *A*. *mirabilis* was sampled at 5, 10 and 20 m, while the rest of species were collected from 10, 20 and 30 m depth. These collection depths represent well the vertical arrangement of these seaweeds at this site (**Fig 1**), where the fucoid *A*. *mirabilis* can grow at depths between 1 and 20 m, and hence be exposed to high solar radiation in summer. The bushy species *D*. *menziesii* normally dominates at intermediate depths between 10 and 20 m, but can also be found at 30 m. *Desmarestia anceps* is abundant between 10 and 30 m. The large kelp-like *H*. *grandifolius*, although can be found at 10 m depth, normally is the dominant organism at depths below 20 m where very low light conditions prevail year around. At 30 m depth, *H*. *gradifolius* practically monopolizes some locations (**Fig 1**). A detailed description of the seaweed zonation at Fildes Bay was recently published [9].

After sampling the specimens were immediately transferred to the laboratory at “Base Julio Escudero” station, where they were kept for a maximum of two days in a cool chamber at a temperature around 2°C with aeration under light:dark cycle (20 h:4 h) for acclimation prior to experimentation. In general fronds (200–300 g) were collected from the middle-distal regions of thalli in order to avoid shaded material (specially in the case of both *Desmarestia* species) and tissues with high epiphyte load (which is common in old specimens of *A*. *mirabilis* and *H*. *grandifolius*).

### Measurement the underwater solar radiation

**Fig 2:**
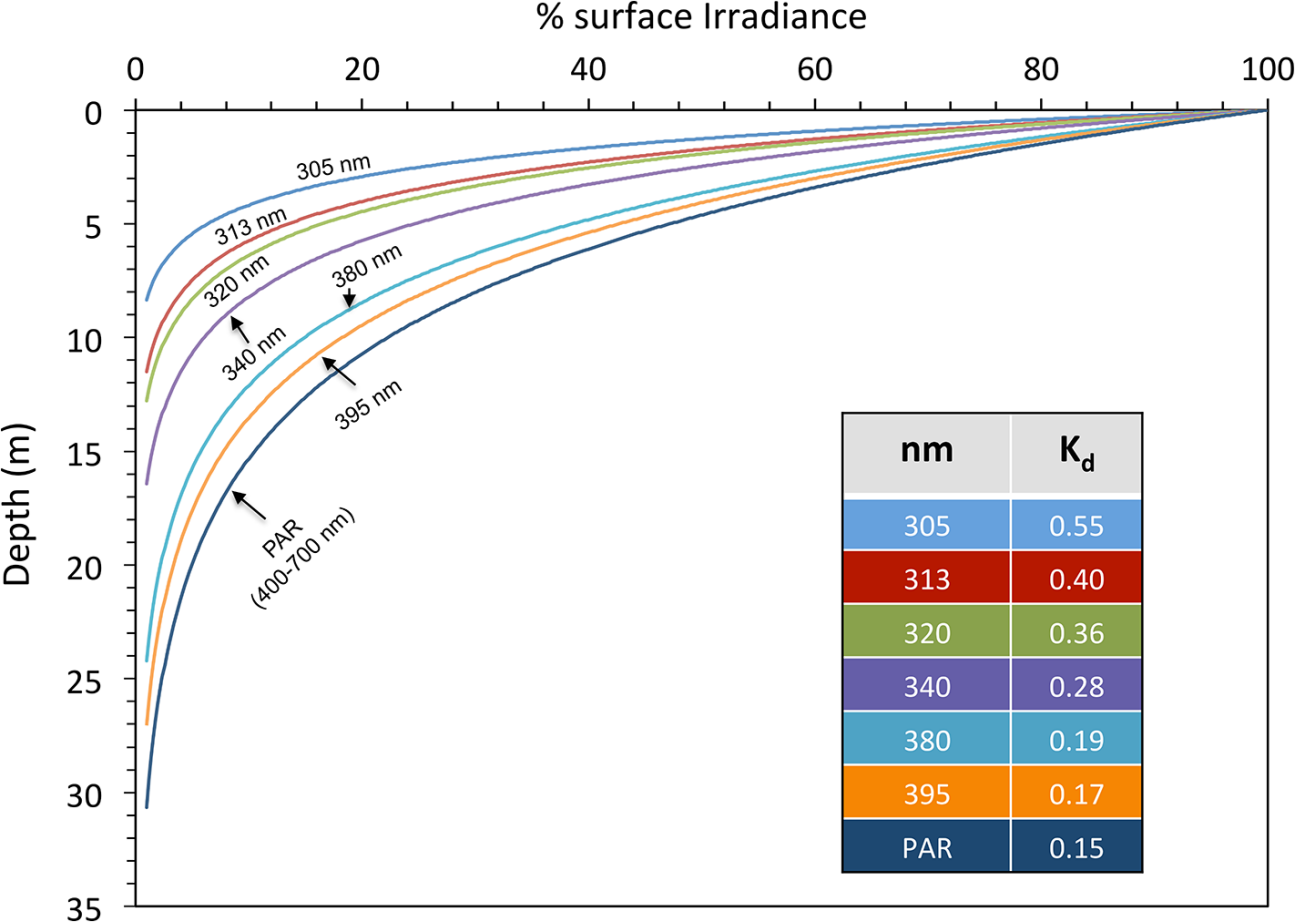
Vertical profile of solar radiation penetration and attenuation coefficients (K_d_ in m^-1^) for different wavelengths of UV-B and UV-A radiation and PAR band (400–700 nm). Values represent average of a representative sunny day at mid-summer (January 2013) at Fildes Bay.

Underwater solar irradiation was measured with a submersible UV profiler radiometer PUV 2500 (Biospherical Instruments Inc., USA) which is designed to measure downwelling (cosine) irradiance, pressure/depth, temperature and fluorescence. UV-B (305 and 313 nm), UV-A (320, 340, 380, and 395 nm) and photosynthetically active radiation (PAR; 400–700 nm) were recorded around noon (12:00 to 14:00 h) in different points of the Fildes Bay. During a sunny day the 1% of UV-B wavelengths (305 and 313 nm) penetrated to 8 and 11 m, with attenuation coefficients (K_d_) of 0.55 and 0.4 m^-1^, respectively. UV-A wavelengths (320, 340, 380 and 395 nm) showed attenuation between 0.36 and 0.17 m^-1^. The broad band of PAR penetrated deeply (1% was close to 31 m) with a K_d_ of 0.15 m^-1^ (**Fig 2**).

### Determination of photosynthetic characteristics

The photosynthetic characteristics of macroalgae from different depths were assessed through electron transport rate (ETR) based photosynthetic versus irradiance (P-I) curves. The algal samples (four replications) were placed into a dark chamber and irradiated with increasing intensities of PAR (up to 370 μmol photon m^-2^ s^-1^) provided by a pulse amplitude modulation fluorometer (PAM 2000, Walz, Germany). ETR was determined by relating effective quantum yield (Φ_PSII_) and the intensity of the actinic irradiance [23]:

$$ETR=\Phi_{PSII}\times E\times A\times 0.5$$

where E is the incident irradiance of PAR and A the thallus absorptance. The factor 0.5 comes from the assumption that four of the eight electrons required to assimilate one CO_2_ molecule are supplied by PSII. Absorptance was measured with a cosine corrected PAR sensor (Licor 192 SB, Lincoln, USA), and calculating the light transmission as:

$$A=1-\frac{E_{t}}{E_{0}}$$

where E_o_ is the incident irradiance and E_t_ the irradiance transmitted beneath the alga. The non-linear function of Jassby and Platt [24] was fitted to the data in order to define the ETR parameters:

$$ETR=ETR_{\max}\times \tanh(\alpha \times \frac{E}{ETR_{\max}})$$

where ETR_max_ is the maximal ETR, tanh the hyperbolic tangent function, α the initial slope of the P-I curve (an indicator of the electron transport efficiency), and E the incident irradiance. Finally, the saturating point for photosynthesis (E_k_) was calculated as the quotient between ETR_max_ and α.

### Experimental exposure to UV radiation

Algal thalli (6–9) of each species were exposed to UV+PAR and PAR treatments for 6 h followed by a 12-h recovery period under dim PAR irradiance, at a temperature of 2°C. The incubation system consisted of a combination of UV (Q-Panel-313 nm and 340 nm fluorescent tubes, Q-Panel Co., Cleveland, OH) and PAR lamps (Daylight, Philips, The Netherlands). Two irradiation treatments were set by use of cut-off filters Ultraphan 295 (Digefra, Munich, Germany; removing wavelengths <295 nm) for UV+PAR treatment, and Ultraphan 395 for PAR treatment (wavelengths <395 nm removed). The irradiance levels in the experimental system were 20 μmol m^-2^ s^-1^ for PAR, 3 W m^-2^ for UV-A and 0.25 W m^-2^ for UV-B. The levels of irradiation were measured with a Li-1400 data logger (LI-COR Biosciences, USA) fitted with a Li-190-S quantum sensor for PAR and two semispherical UV-A/UV-B detectors (Walz, Germany). The experimental levels of UV-B radiation match the ranges measured *in situ* for this locality [9]. After the exposure and recovery phase samples were measured for PAM fluorescence. Following this, the samples were deep frozen in liquid nitrogen and stored dried in silica gel for further biochemical determinations in the laboratory in Valdivia.

### Measurement of chlorophyll fluorescence

The maximum quantum yield (F_v_/F_m_) which indicates the ratio of variable to maximal fluorescence of chlorophyll *a* of photosystem II (PSII) was measured with a fluorometer PAM-2000 in algal samples previously kept in the darkness for 5 min. Inhibition of F_v_/F_m_ was calculated as the percentage decrease between the value measured in the PAR+UV treatment and the value measured in samples exposed to PAR. Similarly, the recovery was estimated by comparing the F_v_/F_m_ values of samples exposed to UV treatment with those obtained after exposure for 12 h to PAR treatment.

### Determination of phlorotannin content

Phlorotannin content was assessed in 4–6 replicates with the Folin-Ciocalteu method as described in [25]: the soluble fraction was extracted from 10 mg dry material homogenized with liquid nitrogen in a mortar. The extracts were mixed with 1 ml acetone (70%) and maintained overnight at 4°C under shaking. After centrifugation (4000 rpm, 10 min) 50 μl of supernatant was added to a solution containing 250 μl of dH_2_O, 200 μl of 20% NaCO_3_ and 100 μl of 2 N Folin-Ciocalteu reagent. The extracts were incubated at room temperature in the darkness for 45 min, and centrifuged at 5000 rpm for 3 min. The absorbance at 730 nm was finally measured in a Multiskan Spectrum spectrophotometer (Thermo Fisher Scientific Inc., Waltham, MA). The content of phlorotannins was determined using phloroglucinol (SIGMA) as a standard. Based on calibration curves, the phlorotannin contents were expressed in dry weight units.

The insoluble phlorotannin fraction was quantified using a modified alkaline method [26, 27]. The alkaline treatment was repeated four times, and the aliquots of each treatment were analyzed separately. The precipitated aliquot was solubilized using a series of solvents in the following order: methanol, H_2_O, methanol, acetone and diethyl-ether. After drying for 1 h at 60°C, the insoluble residue was resuspended in 800 μl of 1 M aqueous NaOH and stirred for 2.5 h. Samples were centrifuged (3000 rpm, 5 min) and 100 μl aliquots neutralized with 10 μl of H_3_PO_4_.

### Determination of antioxidant activity

The radical scavenging activity was determined through the free radical 2,2-diphenyl-1-picrylhydrazyl (DPPH) scavenging method [28, 29]. The sample extract (22 μL) was solubilized in a solution containing 150 μM solution of 80% methanol-DPPH* and the absorbance was measured at 520 nm using Trolox (6-hydroxy-2,5,7,8-tetramethylchroman-2-carboxylic acid) as a standard. The antioxidant activity was defined as μmol Trolox equivalent on dry weight basis. The changes in the activities were calculated as percentage by comparing the initial values (AC_c_) with the values after treatment (AC_t_):

%Radicalscavenging=(ACt-ACc)ACc-1×100.

### Statistical analysis

The variation of physiological responses (P-I curve parameters as well as the photosynthetic and biochemical responses to UV exposure and recovery) along the depth gradient was compared using one-way ANOVA. The assumptions homogeneity of variances and normal distribution were examined using the Levene and Shapiro-Wilk W tests, respectively. The Tukey’s HSD post hoc analysis was used when differences in means were detected.

The similarity analysis (ANOSIM) was used to observe differences between depths based on the suite of physiological variables related to light use characteristics (ETR_max_, α, E_k_) and anti-stress mechanisms (soluble-and- insoluble phlorotannins, the soluble/insoluble phlorotannin ratio, antioxidant capacity and F_v_/F_m_). We applied Similarity Percentage (SIMPER) routines to verify the contribution of each variable to the similarities or differences for each depth group. The spatial distribution of samples was visualized using non-Metric Multi-Dimensional Scaling (nMDS) [30]. The similarity matrix for MDS and ANOSIM was obtained through the Euclidean distance with 999 permutations in the case of ANOSIM. Analyses were conducted in PRIMER v6 + PERMANOVA [31].

## Results

### Depth patterns

**Fig 3:**
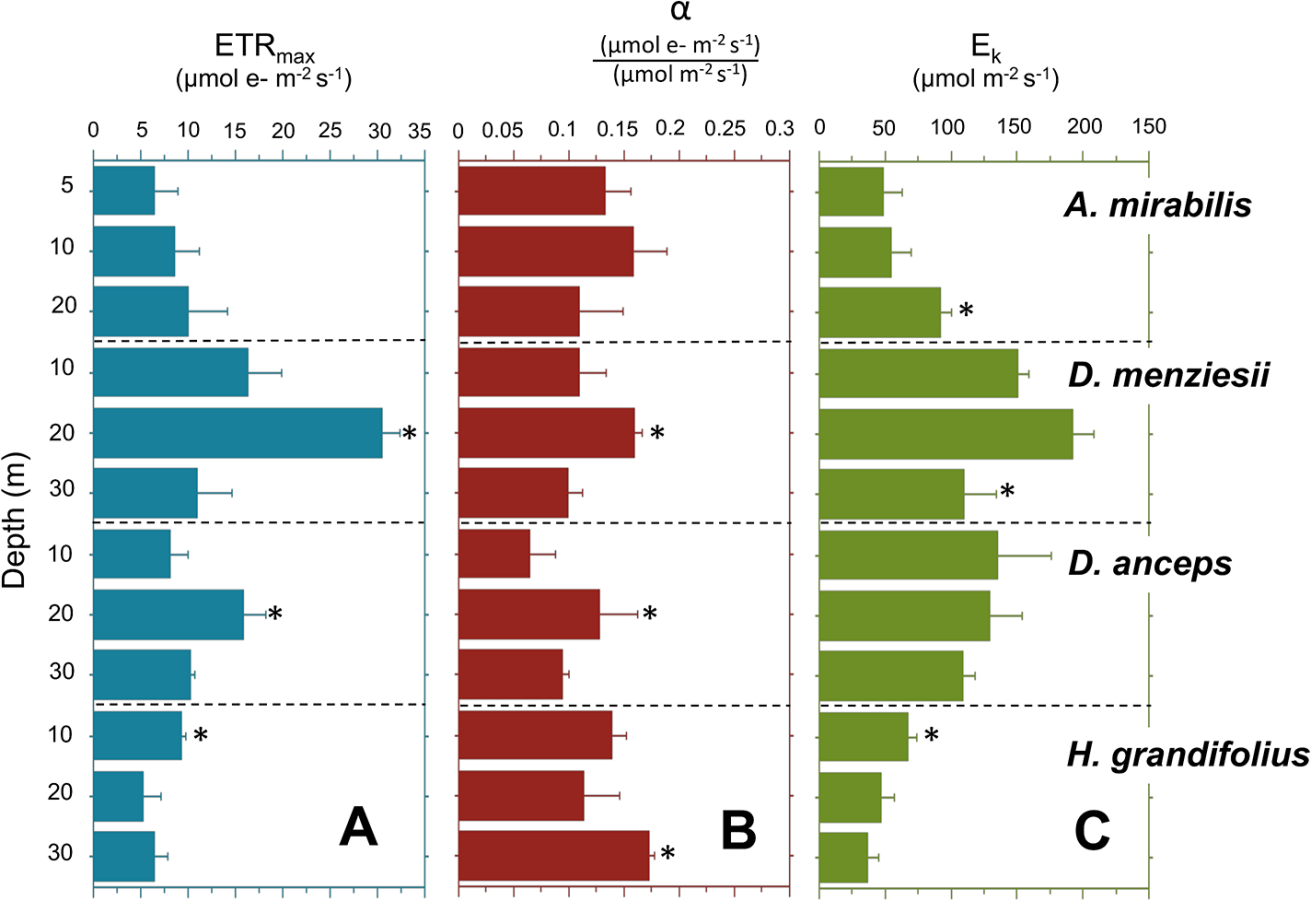
Variation in photosynthetic parameters (A) ETR_max_, (B) initial slope and (C) E_k_ calculated from electron transport rate based P–E curves of four Antarctic brown collected from three different depths at Fildes Bay. Values are means ± S.D., n = 4. Asterisks indicate significant difference (p< 0.05) after ANOVA and Tukey HSD post-hoc test.

P-I curve based parameters measured in the different species varied along the depth gradient (**Fig 3**). The highest ETR_max_ values (30 μmol e- m^-2^ s^-1^; < 0.05) were measured in *D*. *mensiezii* collected from 20 m. Only in *H*. *grandifolius* clear depth patterns were observed, with individuals at shallow habitat (10 m) showing a higher ETR_max_ and E_k_ values while in individuals from deeper site (30 m) higher ∝ value was detected (**Fig 3**). In contrast, *A*. *mirabilis* showed the highest E_k_ value at the deepest site. Considering the three depth ranges and the four species, the average values of ETR_max_ at 5–10, 10–20 and 20–30 m averaged 10.1, 14.4 and 9.4 μmol e- m^-2^ s^-1^, respectively. Mean values of E_k_ and α between 114 and 87 (μmol e- m^-2^ s^-1^) (m^-2^ s^-1^)^-1^ and 0.11 and 0.12 did not vary significantly with depth (**Table A in S1 Table**).

**Fig 4:**
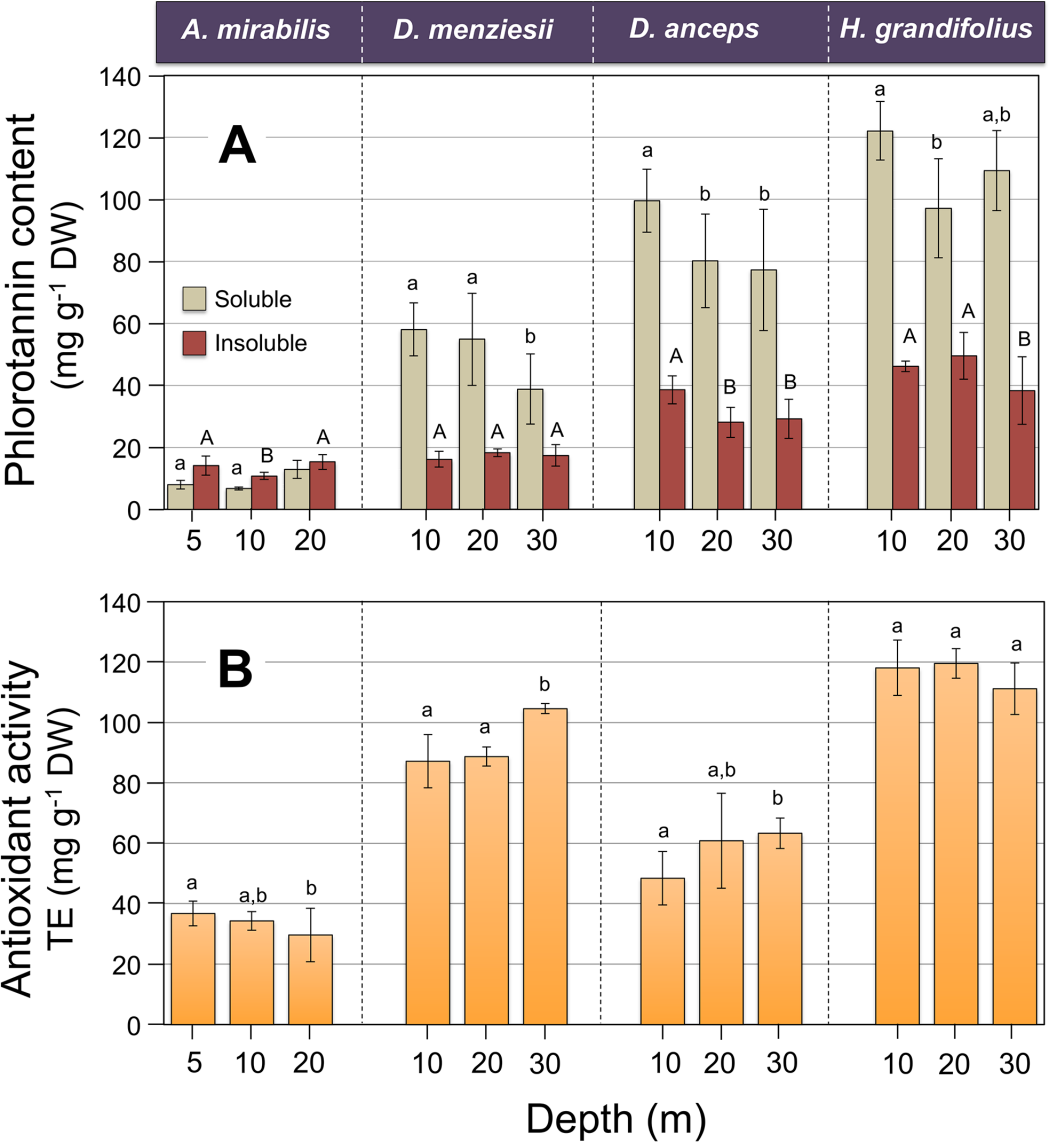
Vertical variation in contents of soluble and insoluble phlorotannins (A) and antioxidant capacity (B) determined in extracts of four species of brown algae collected at three different depths at Fildes Bay. Values are means ± S.D., n = 6–9. Similar letters connect homogeneous means following ANOVA and Tukey HSD post-hoc test.

The concentrations of soluble phlorotannins ranged between 6.4 and 122.1 mg g^-1^ DW, with *Ascoseira mirabilis* allocating the lowest values while *Himantothallus grandifolius* was the species exhibiting the highest concentrations (**Fig 4A**). Similar tendency was found for the insoluble fraction, which ranged between 10.8 and 49.5 mg g^-1^ DW. Along the depth gradient, *D*. *menziesii*, *D*. *anceps* and *H*. *grandifolius* showed the highest contents of the soluble phlorotannins in the samples collected from 10 m (p>0.05, one-way ANOVA, Tukey HSD; **Fig 4A, Table B in S1 Table**). For the insoluble phlorotannins, although some differences between depths were detected within each species, no clear patterns could be recognized. Only in *A*. *mirabilis* the insoluble phlorotannins exceeded the soluble fraction. In general, in the Desmarestiales the ratio between soluble and insoluble phlorotannins varied between 2 and 3.7 and changes with depth could not be detected (**Fig 4A**). The antioxidant capacity in the studied species varied between 29.6 and 119.6 TE (mg g^-1^ DW), with *Ascoseira mirabilis* exhibiting the lowest values. Along the depth gradient, no clear differences in ROS scavenging capacity were detected (**Fig 4B; Table C in S1 Table**). Only in *D*. *menziesii* and *D*. *anceps*, antioxidant activity measured in algae collected at 30 m depth was significantly higher compared to 20 and 10 m (>0.05, one-way ANOVA, Tukey HDS, **Fig 4B**).

### Responses to radiation UV

**Fig 5:**
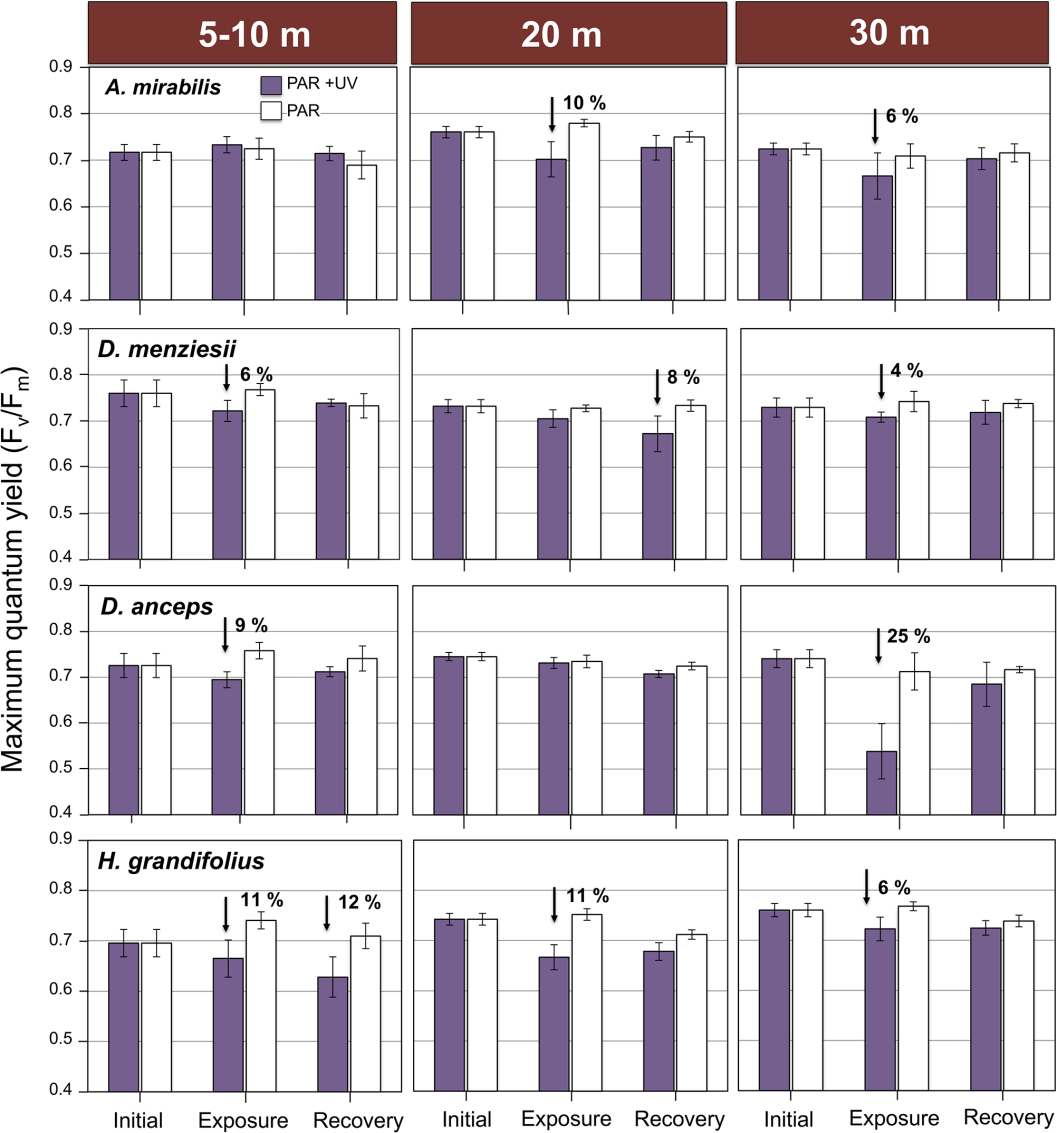
Effect of UV radiation on the maximal quantum yield of fluorescence (F_v_ ⁄ F_m_) of four Antarctic algae collected to different depths. Algae were exposed for 6 h to PAR+UV-A+UV-B (PAR + UV) and PAR conditions (20 μmol m^-2^ s^-1^ for PAR, 3 W m^-2^ for UV-A and 0.25 W m^-2^), and returned to control culture conditions under dim PAR for 12 h (recovery). Initial is the time cero before the 6 h exposure to UV radiation. Values are means ± S.D., n = 9. The significant decrease in photosynthesis in the UV treatment relative to PAR (ANOVA and Tukey HSD post hoc test) is indicated.

Results of F_v_/F_m_ from 6-h UV exposure indicated, overall, high UV tolerance of the studied algae. In *A*. *mirabilis* and *D*. *menziesii*, the most UV tolerant species, F_v_/F_m_ decreased between 0 and 10% in UV relative to PAR treatment (**Fig 5**). Decreases in photosynthesis in *D*. *anceps* were highly variable in relation with the collection depth: at 20 m photosynthesis practically did not decrease after 6 h exposure to UV radiation, but in algae collected at 10 and 30 m, F_v_/F_m_ was inhibited by 9 and 25%, respectively (>0.05; **Fig 5**). In *H*. *grandifolius*, F_v_/F_m_ decreased 6% in algae collected at 30 m and 11% in algae from 20 m depth (>0.05, **Fig 5**). In all the studies species, irrespective of their collection depth there was a tendency of a full recovery of photosynthesis after exposure for 12 h to dim light (**Fig 5**). The factorial ANOVA indicated a significant interaction of depth and UV treatment in *A*. *mirabilis* and *D*. *anceps*, while in *D*. *menziesii* and *H*. *grandifolius*, only single effects of both factors on the variability of F_v_/F_m_ were observed (p> 0.01, two-way ANOVA; **Table A in S2 Table**).

**Fig 6:**
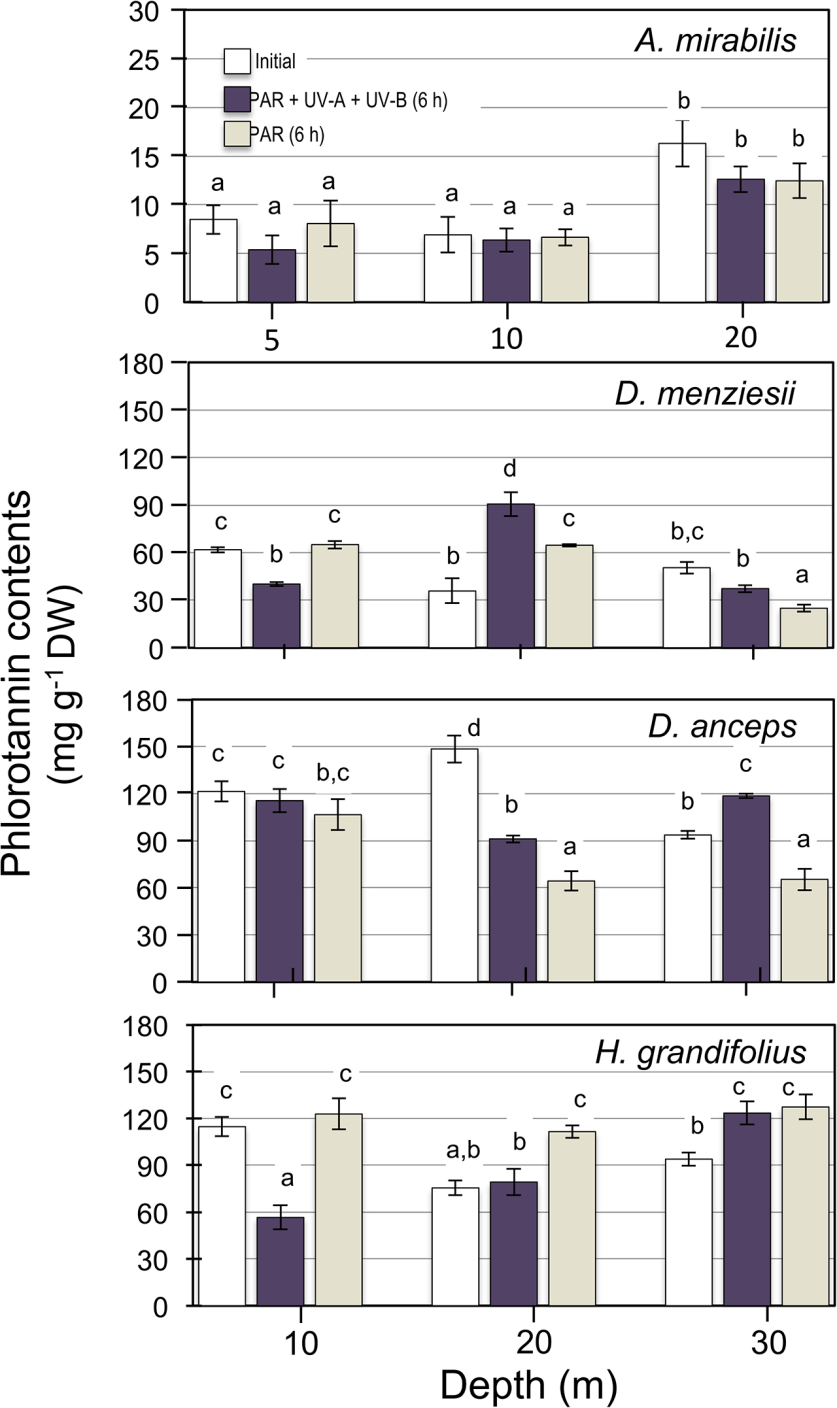
Effect of UV exposure on the concentration of phlorotannins measured in four brown algae collected at different depth in Fildes Bay. Algae were exposed for 6 h to PAR+UV-A+UV-B and PAR conditions (20 μmol m^-2^ s^-1^ for PAR, 3 W m^-2^ for UV-A and 0.25 W m^-2^). Initial is the time cero before the 6 h exposure to UV radiation. Values are means ± S.D., n = 3. Similar letters connect homogeneous mean groups for each species after two-way ANOVA and Tukey’s HSD.

The responses of soluble phlorotannins to UV radiation did not shown clear depth patterns, but there were marked differences between species (**Fig 6**). In *A*. *mirabilis*, no effects of UV exposure were detected in algae from 5 and 10 m depth (p>0.05). However, due to that in algae collected at 20 m phlorotannin contents were higher than at 5 and 10 m (p<0.05, Tukey HDS), the factorial ANOVA indicated a significant interaction between depth and UV treatment (**Table B in S2 Table**). In *D*. *menziesii* from 10 m and *H*. *grandifolius* from 10 and 20 m, phlorotannins significantly decreased (28–54%) when exposed to UV radiation (p<0.05; **Fig 6**). In contrast, samples of *D*. *menziesii* from 20 m and *D*. *anceps* from 30 m showed an increase in phlorotannin contents after UV exposure (p<0.05). Similar as in the fucoid *A*. *mirabilis*, the two-way ANOVA indicated a significant interactive effect (p< 0.001) of depth and type of UV treatment on the variability of phlorotannins in the three species of Desmarestiales (**Table B in S2 Table**).

**Fig 7:**
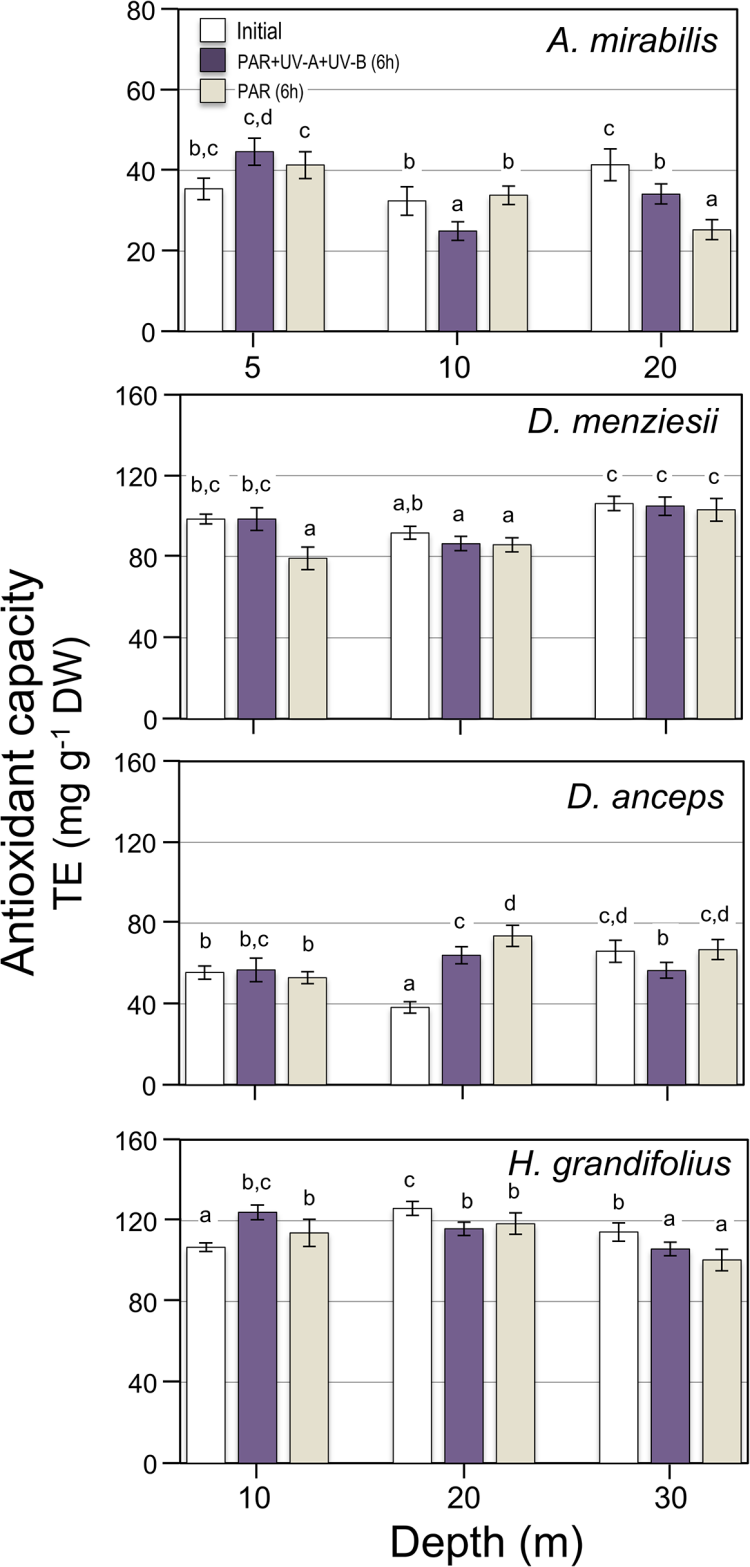
Effect of UV exposure on the antioxidant capacity measured in four brown algae collected at different depth in Fildes Bay. Algae were exposed for 6 h to PAR+UV-A+UV-B and PAR conditions (20 μmol m^-2^ s^-1^ for PAR, 3 W m^-2^ for UV-A and 0.25 W m^-2^). Initial is the time cero before the 6 h exposure to UV radiation. Values are means ± S.D., n = 3. Similar letters connect homogeneous mean groups for each species after two-way ANOVA and Tukey’s HSD.

The antioxidant capacity varied between 24 TE (mg g^-1^ DW) in *A*. *mirabilis* and 126 TE (mg g^-1^ DW) in *H*. *grandifolius* and was not affected by exposures to UV radiation (**Fig 7**), with an interaction between the factors (**Table C in S2 Table**).

### Multivariate analysis and variable relationships

**Fig 8:**
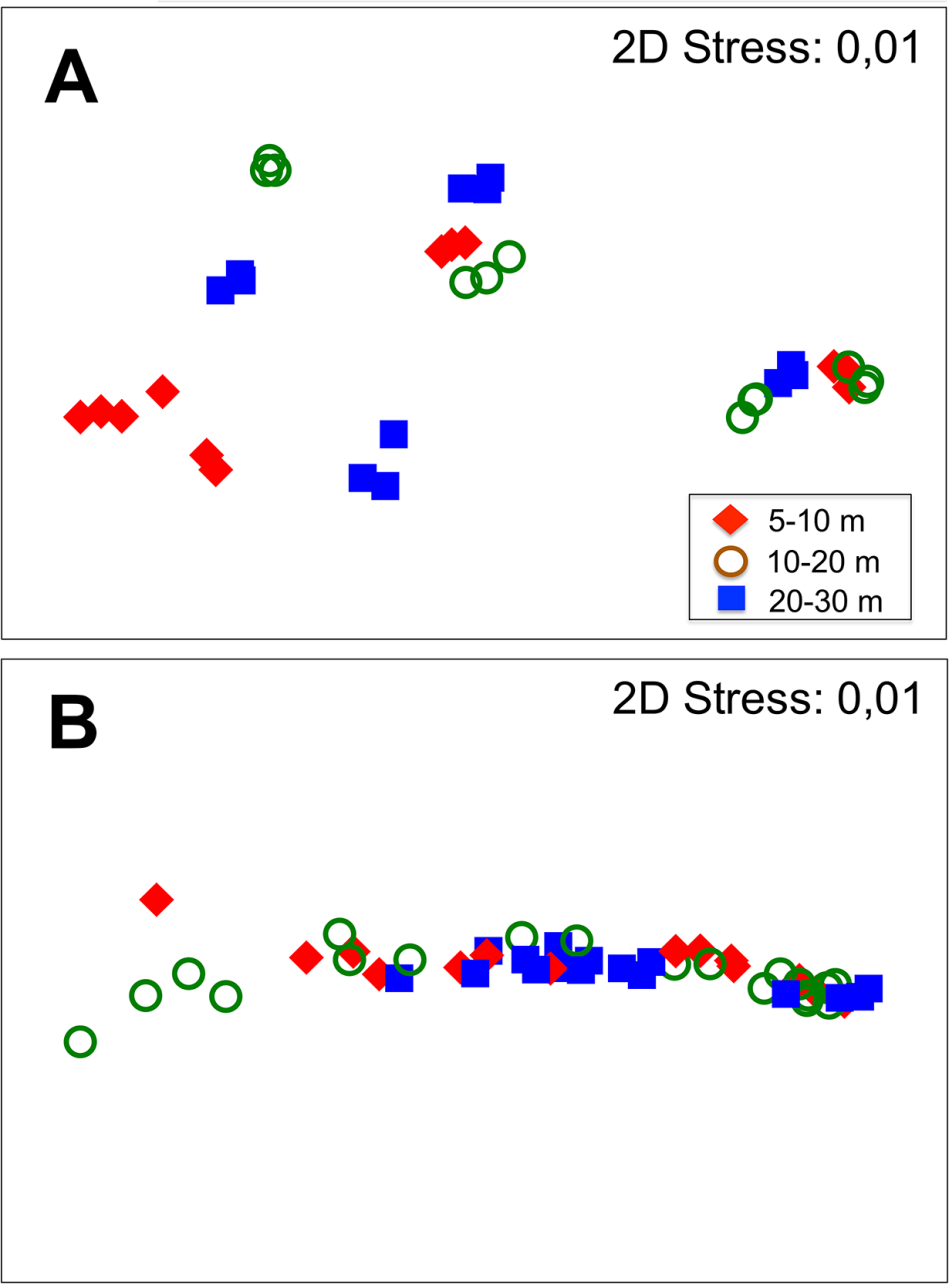
Multi-dimensional Scaling Ordinations (MDS) of physiological profiles associated with light use characteristics (A) and UV stress tolerance parameters (B) in relation with depth.

MDS ordination revealed differences along depth when photosynthetic characteristics (**Fig 8A**) and stress tolerance (**Fig 8B**) were regarded. The SIMPER analysis indicated that the response of the photosynthetic characteristics was similar between 5–10 and 10–20 m and different at 20–30 m depth. Light requirements for photosynthesis (E_k_) contributed between 96 and 99% to the total dissimilarities between depths, while ETR_max_ varied between 3.8 y 0.8% (**Table 1**). The response of the variables related with stress tolerance (phlorotannin content, antioxidant activity) was similar at 5–10 and 20–30 m and different at 10–20 m. According to the SIMPER analysis, the antioxidant activity and soluble phlorotannins were the variables with the highest contribution to the observed similarity/dissimilarity among samples. The antioxidant activity accounted by a 79% of the variability at 5–10 m, while at 10–20 and 20–30 m, its contribution decreased to values close 22%. In contrast, the contribution of the insoluble phlorotannins was 56 and 68% in the 10–20 and 20–30 m decreasing to 10% at 5–10 m depth (**Table 1**).

**Table 1:** SIMPER analysis of different photosynthetic and biochemical variables measured in four Antarctic brown algae that contribute to the dissimilarity across different depth strata. Photosynthetic parameters: maximal photosynthesis measured as ETR values; saturation irradiance for photosynthesis (E_k_); maximum electron transport rate (ETR_max_); initial slope of the ETR-I curve (α); maximum quantum yield (F_v_/F_m_).

Photosynthetic parameters related with light adaptation		Physiological variables related with stress tolerance	
Variable	%	Variable	%
**5–10 m**			
E_k_	99.17	Antioxidant capacity	78.68
ETR_max_	0.83	Soluble phlorotannins	11.30
α	0	Insoluble phlorotannins	9.66
		Ratio Sol/Insol	0.17
		F_v_/F_m_	0
**10–20 m**			
E_k_	98.40	Antioxidant capacity	21.81
ETR_max_	1.60	Soluble phlorotannins	56.45
α	0	Insoluble phlorotannins	20.51
		Ratio Sol/Insol	1.23
		F_v_/F_m_	0
**20–30 m**			
E_k_	96.19	Antioxidant capacity	23.25
ETR_max_	3.81	Soluble phlorotannins	68.37
α	0	Insoluble phlorotannins	8.23
		Ratio Sol/Insol	0.15
		F_v_/F_m_	0

**Fig 9:**
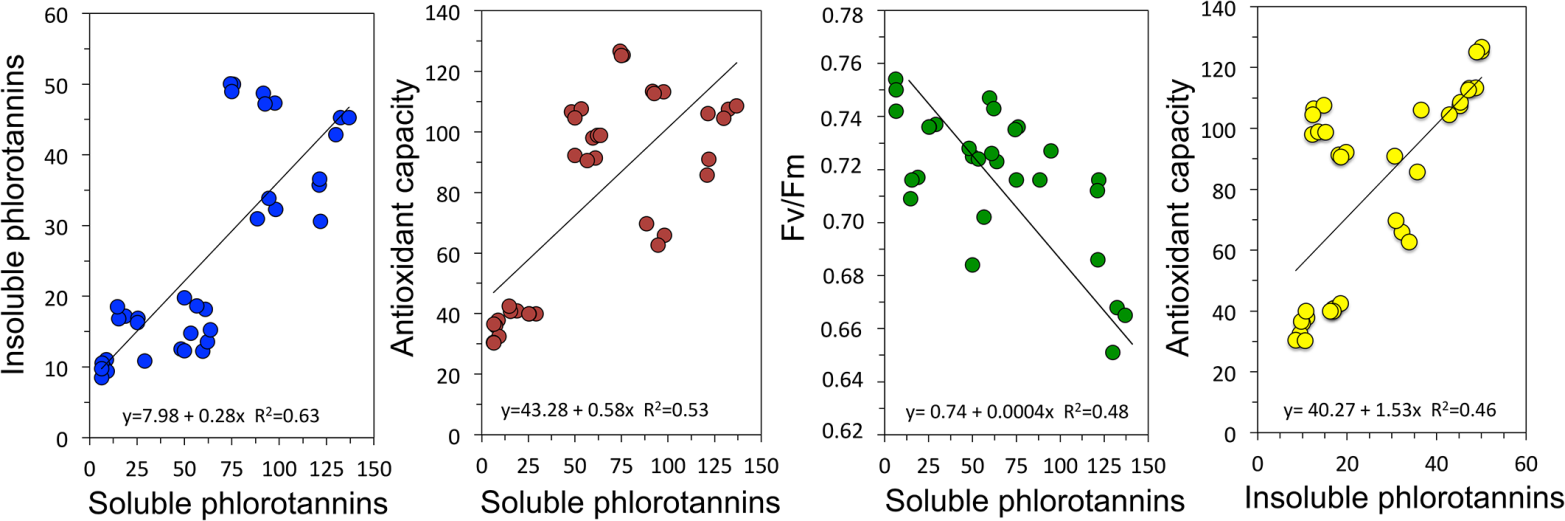
Relationship between different physiological variables determined in four species of endemic Antarctic brown algae collected at three different depths.

The Spearman correlation analysis indicated that the soluble phlorotannins were significantly and positively related with the insoluble phlorotannins (R^2^ = 0.63), while the antioxidant activity of the extracts were positively correlated with both phlorotannin fractions (R^2^ = 0.53 and 0.46, respectively). Finally, F_v_/F_m_, an indicator of potential for light absorption and efficiency, was higher when phlorotannins were at lowest (**Fig 9**).

## Discussion

### Photosynthetic light demand

The conspecific individuals of all studied species growing at depths across a zonation range between 5 and 30 m showed variability in their photosynthetic characteristics, especially the light demands for photosynthesis (α and E_k_). However, no clear patterns in relation with the depth were observed. In general, light requirements for photosynthesis are in the range of irradiance levels measured in summer at the study site.

The capacity of macroalgae to set their physiological limits to the available light gradients is probably the most important factor explaining the depth distribution of these organisms [32]. Although vertical distribution of Antarctic seaweeds in the subtidal zone cannot be only explained by light limitation, and other factors such as substrate characteristics, strong perturbation due to anchor ice or herbivory can become relevant [3, 33, 34], light supports primary productivity at the lower limit of seaweed colonization [35, 36]. Based on the light profiles measured at Fildes Bay during summer, it is reasonable to argue that macroalgae were not light limited as 1% of average PAR reached 30 m depth. This depth coincides with the limit for positive carbon balance, and hence can be regarded as a critical depth for primary productivity estimated for large endemic Antarctic brown algae [10]. This is probably why large polar brown algae (e.g. Laminariales and Desmarestiales) show comparable morpho-functional processes based on a seasonal synchronization of photosynthesis and biomass formation (reviewed in [15]). Although statistical differences between depths in the light demand characteristics were detected, no clear patterns with depth gradient could be outlined, except for *Himantothallus grandifolius*. However, the multivariate analysis using SIMPER revealed that E_k_ is the most relevant parameter that define shade adaptation in these species, accounting by > 96% of the dissimilarity associated with depth groups. Additionally, the null impact of the phtotosynthetic efficiency (α) as a reliable predictor of depth differences in photosynthesis reinforced the findings that Antarctic algae show low potential acclimation to different light fields [10, 13]. Thus, it is now clear that high photosynthetic efficiency and relatively low light demands for saturation of photosynthesis along their entire depth distribution allow these species to photosynthesize at high rates during summer, storing photoassimilates in surplus as carbohydrates, and finally remobilizing them in spring/winter to minimize carbon accretion due to growth as was reported time ago using ^14^C and O_2_ evolution probes [10, 14]. Similar patterns had been reported for the deep Arctic kelp *Laminaria solidungula* from the turbid Alaskan Beaufort Sea, in which high photosynthetic efficiency and low light requirements for photosynthesis remain relatively unaltered with season because this species only receives 45 mol photons m^-2^ year^-1^ (< 1% of total surface PAR), probably the lowest photon flux of any deep water kelp [37]. Under these conditions frond elongation in this species relies on the remobilization and the use of photosynthates stored during the short summer: a strategy that permits the alga to replenish around 90% of the total carbon lost in winter [38]. In contrast, during the Antarctic summer, the water column at Fildes Bay is relatively clear (see **Fig 2**) and deeper algae such as *H*. *grandifolius*, in virtue of their E_k_ values between 30 and 40 μmol m^-2^ s^-1^, can actively photosynthesize at depths between 20 and 30 m depth where incident irradiances could be close to 3 and 4% of the maximal water surface levels recorded at King George Island [9, 39].

In terms of their bio-optical properties, the studied endemic Antarctic brown algae, which display a multilayered, thicker morphology, can be regarded as optically “opaque” or “black” and hence are characterized by higher light absorptance (normally > 0.8) than filamentous or sheet-like forms [40, 41]. These attributes have important consequences for depth distribution of algae and their capacity to exploit the light field prevailing at depth habitats, especially if one considers the absorption spectra [42]. Due to differential attenuation, at 10 m depth incident solar radiation at Fildes Bay is strongly depleted, especially at 400–450 and 600–700 nm where chlorophylls show their absorption peaks [9]. Thus, ticker brown algae attaining heterogeneous light paths within the thallus increasing backscatter of light and high contents in accessory pigments such as fucoxanthin and chl *c* enhancing photon capture, are favored in these conditions [43]. The bio-optical traits could be regarded as reliable functional parameters to explain the prevalence of large brown algae at deeper locations in various costal systems worldwide [41,44,45]. Based on these considerations, not only a positive carbon balance at very low light but also a putative high light trapping capacity and photochemical efficiency of endemic Antarctic brown algae, are probably key physiological factors that set their lower limits of vertical distribution. In scenarios of climate change, such photobiological features can prove be advantageous, e.g. during increased water turbidity due to enhanced glacier melting [22] (see below).

### UV tolerance

The remarkable capacity of the strongly shade-adapted endemic Antarctic brown algae to tolerate short-term exposure to UV radiation reflected in low reduction in photosynthesis, high levels of phlorotannins and consistent antioxidant capacity across the whole vertical distribution range highlights a paradoxical feature of these organisms. In contrast to the algae living at deeper subtidal zones, upper limits of many seaweeds are constrained by UV radiation, temperature, and salinity (reviewed in [17]). However, some morpho-functional and bio-optical attributes of Antarctic algae that allow them occupying the whole range of light climates apparently are functional to cope with harmful solar radiation. For example, a thick, multilayered morphology, which associated with enhanced absorption of PAR, also allows these algae minimizing the impact of harmful UV by increasing the thallus cross section and hence attenuating the number of UV quanta reaching target molecules [46,47]. It must be emphasized that UV photoprotection by morphological traits is strongly superimposed by other selective factors related with thallus growth patterns, size scape from grazers, competition for space, etc. [35,41,44].

ANOSIM/SIMPER analysis revealed that the contents of phlorotannins and the antioxidant activity were the most relevant physiological factors that discriminated the groups of algae by depth. Our results confirmed the predictions that phlorotannins are key components of the suite of physiological mechanisms that seaweeds display to respond to environmental stressors, similar as has also been reported in brown algae from other geographical regions [29,48,49]. The constitutively higher levels of soluble phlorotannins, especially in those species dominating depth > 20 m (*D*. *anceps* and *H*. *grandifolius*) compared to levels measured in species that dominate at shallower locations (*A*. *mirabilis* and *D*. *menziesii*) showed a patterns correlated with the antioxidant capacity in response to UV radiation. These differences between species have been outlined previously for different Antarctic brown algae collected from a single depth [9; 20]. However, when using conspecifics of these species inhabiting different depths, such patterns were not found, suggesting that phlorotannin concentration is retained among different populations irrespective of their vertical distribution, similar as photosynthetic characteristics related with shade adaptation. Similarly, either the phlorotannin content or antioxidant activity from different depths did not show relevant variations after UV exposure, suggesting that phlorotannins act as non-inducible photoprotectants, as was previously reported for *D*. *anceps* and *D*. *mensiezii* exposed for several weeks to UV radiation [50]. Taking into account that the algal material collected during this study had low growth activity (normally these algae show the highest rates of thallus elongation in late winter/spring) [14; 51], the high levels of soluble and insoluble phlorotannins could be strongly related with biomass formation processes [52] and not with UV protection. In fact, the levels of cell wall-bound, insoluble phlorotannins were closely related with the soluble fraction, relatively constant across the depths and represented between 20 to 37% of the soluble fraction in algae belonging to the Desmarestiales. In *A*. *mirabilis*, the concentrations of phlorotannins were significantly lower than in Desmarestiales and also the insoluble fraction was relatively similar to the soluble ones. Because soluble phlorotannins are precursors of the insoluble fraction, which has a primary role (structural) of these compounds, our results suggest a complex interplay between various environmental (e.g. UV radiation, temperature, water movement) and intrinsic factors (morphology, growth, phylogeny, etc.). For example, in the sub-Antarctic kelp, *Lessonia spicata* (formerly *L*. *nigrescens*), common at the intertidal systems, soluble phlorotannin are inducible only when algae are elongating their fronds, but not during winter when growth ceases [25]. In other cases, differences in habitat (e.g. wave exposure) can affect the contents of phlorotannins among co-existing species of large sub-Antarctic brown algae [49,53].

### Climate change scenarios

In light of the climate change in Antarctica, the question whether the observed physiological characteristics of endemic brown algae in the context of vertical distribution, i.e. high photosynthetic efficiency, low demands for photosynthesis as well as high potential for UV stress tolerance, will be modified in ecologically significant scales become relevant. Apparently, global warming could have contrasting consequences for coastal primary productivity. Firstly, under a “normal” scenario, a well-developed sea-ice cover acts attenuating light and protects primary producers from UV radiation, and consequently many ecosystem processes will be closely tied to the few months of open water [54,55]. However, in scenarios of enhanced warming, a prolonged duration of ice-free season could increase benthic primary production in species growing at deeper locations [56–58] but also will increase the time span at which the organisms could be exposed to harmful UV radiation, especially species living at shallower sites [9,17]. In Antarctica, brown seaweeds not only are the structuring support of the whole benthic community but also represent a year-round essential carbon sink through production of high biomass with maximum values estimated for the sublittoral of over 10 kg fresh weight m^-2^ [59]. This biomass represents an important contribution to the polar coastal ecosystem as a food source, either as fresh material or as particulate organic matter (POM) [2, 60]. Unfortunately, very few data on biomass and almost none on production rates of benthic primary producers are available for Antarctica (summarized in [11]). Especially in some areas where phytoplankton productivity is very low it has been postulated that seaweeds and benthic microalgae support essentially the heterotrophic community [61].

Increased sediment run-off from melting glaciers is another important factor affecting severely light penetration in Antarctica [21] and opens a second question: how will the physiological adjustments finally affect distributional patterns of seaweeds? In terms of local impact for seaweeds communities, retreating glaciers have two contrasting consequences: new areas in the upper subtidal and intertidal are now accessible for seaweed colonization as has been reported for the glacier caps on King Gorge Island [21,62]. On the other hand, a more turbid water column due to glacier melting lead to local changes in the lower limit for macroalgal photosynthesis, especially due to that algae have to set their metabolic carbon balance several meters upwards [22]. Furthermore, an increased nutrient supply by enhanced river run-off or glacier melting may support an increase in primary productivity of opportunistic species [63], but when high nutrients coincide with sediment instability, high dissolved organic matter, lower water movement and salinity stratification, hypoxia and related phenomena could prevent benthic metabolism [64], whereas a decrease in salinity may counteract this effect. In all, these predictions require validation based on accurate models, which should integrate physiological and ecological responses at different scales [36].

In conclusion, the results of this study indicate that endemic Antarctic brown algae show metabolic prerequisites to inhabit a broad bathymetrical range in virtue of two paradoxically contrasting characteristics: high photosynthetic efficiency and constitutively high levels of phenolic substances (phlorotannins). Due to that these two physiological traits are retained in conspecifics growing at depth ranges between 5–30 m, it could be reasonable to argue that these species are well suited to exploit a variety of light and UV climates. Finally, this has important consequences for the whole benthic community, as the ability of these canopy-forming algae to cope with changing near-shore environmental scenarios can ameliorate the impact on recruitment and development of understory and other associated assemblages.

## Supporting Information

S1 Table Summary of one-way ANOVA results for the differences in photosynthetic characteristics estimated from P-I curves (Table A); soluble and insoluble phlorotannin contents (Table B) and antioxidant capacity (Table C) measured in four Antarctic brown algae collected at three depths each. Significances: * = p<0.05; ** = p< 0.01; *** = p>0.001.
(DOCX)

S2 Table Summary of two-way ANOVA results for the effect of depth and UV treatment on the maximal quantum yield (F_v_/F_m_) (Table A); contents of soluble phlorotannins (Table B) and antioxidant activity (Table C) measured in four Antarctic brown algae collected to three depths each. Significances: * = p<0.05; ** = p< 0.01; *** = p>0.001.
(DOCX)
