# Supplementary material for: Lack of Physiological Depth Patterns in Conspecifics of Endemic Antarctic Brown Algae: A Trade-Off between UV Stress Tolerance and Shade Adaptation?
Source: PLoS One. 2015 Aug 7;10(8):e0134440. doi: 10.1371/journal.pone.0134440 (PMC4529099; doi:10.1371/journal.pone.0134440)
Supplement: S1 Table — Significances: * = p<0.05; ** = p< 0.01; *** = p>0.001. (DOCX) [file pone.0134440.s001.docx]

**S1 Table**. Summary of one-way ANOVA results for the differences in photosynthetic characteristics estimated from P-I curves (**Table A**); soluble and insoluble phlorotannin contents (**Table B**) and antioxidant capacity (**Table C**) measured in four Antarctic brown algae collected at three depths each. Significances: * = p<0.05; ** = p< 0.01; ***= p>0.001.

**Table A**

| Species | ETRmax | | | Initial slope (α) | | | Satutartion point (E_k_) | | |
| --- | --- | --- | --- | --- | --- | --- | --- | --- | --- |
| Source of variation | MS | d.f. | F | MS | d.f. | F | MS | d.f. | F |
| *A. mirabilis* | | | | | | | | | |
| Intercept | 836.9 | 1 | 81.4*** | 0.212 | 1 | 205.6*** | 50644.7 | 1 | 293.0*** |
| Depth | 12.4 | 2 | 1.2 ns | 0.002 | 2 | 2.3 ns | 2176.1 | 2 | 12.6** |
| Error | 10.3 | 9 |  | 0.001 | 9 |  | 172.8 | 9 |  |
| *D. menziesii* | | | | | | | | | |
| Intercept | 4009.3 | 1 | 423.7*** | 0.161 | 1 | 701.4*** | 216572.2 | 1 | 724.7*** |
| Depth | 401.6 | 2 | 42.5*** | 0.004 | 2 | 17.5** | 4966.7 | 2 | 16.6** |
| Error | 9.4 | 24 |  | 0.001 | 8 |  | 298.9 | 7 |  |
| *D. anceps* |  |  |  |  |  |  |  |  |  |
| Intercept | 1673.2 | 1 | 506.3*** | 0.116 | 1 | 181.7*** | 199115.6 | 1 | 257.9*** |
| Depth | 73.9 | 2 | 22.4** | 0.005 | 2 | 7.1* | 797.7 | 2 | 1.0 ns |
| Error | 3.3 | 10 |  | 0.001 | 10 |  | 772.0 | 10 |  |
| *H. grandifolius* | | | | | | | | | |
| Intercept | 662.4 | 1 | 301.7*** | 0.272 | 1 | 496.2*** | 34133.1 | 1 | 430.1*** |
| Depth | 20.0 | 2 | 9.1* | 0.004 | 2 | 7.8** | 981.7 | 2 | 12.4** |
| Error | 2.2 | 18 |  | 0.001 | 11 |  | 79.3 | 11 |  |

**Table** **B**

| Species | | Soluble | | | | Insoluble | | | | Ratio sol/insol | | |  |
| --- | --- | --- | --- | --- | --- | --- | --- | --- | --- | --- | --- | --- | --- |
| Source of variation | MS | | d.f. | F | MS | | d.f. | F | MS | | d.f. | F |  |
| *A. mirabilis* | | | | | | | | | | | | |  |
| Intercept | 2310.8 | | 1 | 651.4*** | 4838.2 | | 1 | 879.1*** | 12.2 | | 1 | 457.5*** |  |
| Depth | 103.0 | | 2 | 29.0*** | 48.6 | | 2 | 8.8 ns | 0.2 | | 2 | 8.0** | |
| Error | 3.5 | | 24 |  | 5.5 | | 24 |  | 0.03 | | 24 |  | |
| *D. menziesii* | | | | | | | | | | | | | |
| Intercept | 67027.1 | | 1 | 384.4*** | 8058.7 | | 1 | 1218.3*** | 244.1 | | 1 | 183.8*** | |
| Depth | 890.6 | | 2 | 5.1* | 10.0 | | 2 | 1.5 ns | 0.8 | | 2 | 0.6 ns | |
| Error | 174.4 | | 24 |  | 6.6 | | 24 |  | 1.3 | | 24 |  | |
| *D. anceps* |  | |  |  |  | |  |  |  | |  |  | |
| Intercept | 160129.0 | | 1 | 367.9*** | 20569.3 | | 1 | 922.6*** | 155.8 | | 1 | 330.9*** | |
| Depth | 2030.4 | | 2 | 4.6* | 1261.2 | | 2 | 56.5*** | 0.5 | | 2 | 1.1 ns | |
| Error | 435.2 | | 24 |  | 22.3 | | 24 |  | 0.4 | | 24 |  | |
| *H. grandifolius* | | | | | | | | | | | | | |
| Intercept | 236039.9 | | 1 | 901.6*** | 53836.4 | | 1 | 1327.9*** | 137.5 | | 1 | 430.1*** | |
| Depth | 1333.2 | | 2 | 5.1* | 298.1 | | 2 | 7.3** | 3.3 | | 2 | 12.4 ns | |
| Error | 261.8 | | 18 |  | 40.5 | | 24 |  | 1.1 | | 18 |  | |

**Table C**.

| Species | |  | | | |
| --- | --- | --- | --- | --- | --- |
| Source of variation | MS | | d.f. | F |  |
| *A. mirabilis* |  | |  |  |  |
| Intercept | 30432.7 | | 1 | 861.8*** |  |
| Depth | 117.5 | | 2 | 3.3 ns |  |
| Error | 35.3 | | 24 |  |  |
| *D. menziesii* |  | |  |  |  |
| Intercept | 235937.4 | | 1 | 7903.2*** |  |
| Depth | 835.8 | | 2 | 27.9*** |  |
| Error | 29.9 | | 24 |  |  |
| *D. anceps* |  | |  |  |  |
| Intercept | 89382.6 | | 1 | 760.6*** |  |
| Depth | 573.7 | | 2 | 4.8* |  |
| Error | 117.5 | | 24 |  |  |
| *H. grandifolius* |  | |  |  |  |
| Intercept | 365013.6 | | 1 | 6043.0*** |  |
| Depth | 180.2 | | 2 | 2.9 ns |  |
| Error | 60.4 | | 24 |  |  |
