# Supplementary material for: Lack of Physiological Depth Patterns in Conspecifics of Endemic Antarctic Brown Algae: A Trade-Off between UV Stress Tolerance and Shade Adaptation?
Source: PLoS One. 2015 Aug 7;10(8):e0134440. doi: 10.1371/journal.pone.0134440 (PMC4529099; doi:10.1371/journal.pone.0134440)
Supplement: S2 Table — Significances: * = p<0.05; ** = p< 0.01; *** = p>0.001. (DOCX) [file pone.0134440.s002.docx]

**S2 Table**. Summary of two-way ANOVA results for the effect of depth and UV treatment on the maximal quantum yield (F_v_/F_m_) (**Table A**); contents of soluble phlorotannins (**Table B**) and antioxidant activity (**Table C**) measured in four Antarctic brown algae collected to three depths each. Significances: * = p<0.05; ** = p< 0.01; ***= p>0.001.

**Table A**

| Source of variation | d.f. | MS | F |
| --- | --- | --- | --- |
| *A. mirabilis* | | | |
| Intercept | 1 | 77.333 | 58401.0*** |
| Depth | 2 | 0.017 | 13.4*** |
| UV Treatment | 2 | 0.012 | 9.1*** |
| Depth x UV Treatment | 4 | 0.007 | 5.2** |
| Error | 66 | 0.001 |  |
| *D. menziesii* |  |  |  |
| Intercept | 1 | 81.680 | 193111.2*** |
| Depth | 2 | 0.007 | 18.3*** |
| UV Treatment | 2 | 0.011 | 27.8*** |
| Depth x UV Treatment | 4 | 0.001 | 1.1 ns |
| Error | 69 | 0,001 |  |
| *D. anceps* |  |  |  |
| Intercept | 1 | 75.387 | 74249.1*** |
| Depth | 2 | 0.105 | 103,5*** |
| UV Treatment | 2 | 0.048 | 47.9*** |
| Depth x UV Treatment | 4 | 0.033 | 32.5*** |
| Error | 70 | 0.001 |  |
| *H. grandifolius* |  |  |  |
| Intercept | 1 | 81.861 | 57377.3*** |
| Depth | 2 | 0.020 | 14.4*** |
| UV Treatment | 2 | 0.036 | 25.5*** |
| Depth x UV Treatment | 4 | 0.002 | 1.78 ns |
| Error | 70 | 0.001 |  |

**Table B**

| Source of variation | d.f. | MS | F |
| --- | --- | --- | --- |
| *A. mirabilis* | | | |
| Intercept | 1 | 2175.1 | 2268,6*** |
| Depth | 2 | 142.6 | 148,7*** |
| UV Treatment | 2 | 13.9 | 14,5*** |
| Depth x UV Treatment | 4 | 5.3 | 5,6** |
| Error | 18 | 0.9 |  |
| *D. menziesii* |  |  |  |
| Intercept | 1 | 71847.7 | 6992,2*** |
| Depth | 2 | 1401.5 | 136,4*** |
| UV Treatment | 2 | 177.7 | 17,2*** |
| Depth x UV Treatment | 4 | 1860.6 | 181,1*** |
| Error | 18 | 10.2 |  |
| *D. anceps* |  |  |  |
| Intercept | 1 | 171489.6 | 4898,7*** |
| Depth | 2 | 15620.0 | 446,2*** |
| UV Treatment | 2 | 1560.1 | 44,6*** |
| Depth x UV Treatment | 4 | 618.2 | 17,7*** |
| Error | 18 | 35.0 |  |
| *H. grandifolius* |  |  |  |
| Intercept | 1 | 261393.3 | 12688,4*** |
| Depth | 2 | 1864.9 | 90,5*** |
| UV Treatment | 2 | 4664.9 | 226,4*** |
| Depth x UV Treatment | 4 | 3040.3 | 147,6*** |
| Error | 18 | 20.6 |  |

**Table C**

| Source of variation | d.f. | MS | F |
| --- | --- | --- | --- |
| *A. mirabilis* | | | |
| Intercept | 1 | 32689.3 | 5866.7*** |
| Depth | 2 | 238.6 | 42.8*** |
| UV Treatment | 2 | 19.9 | 3.5* |
| Depth x UV Treatment | 4 | 155.4 | 27.9*** |
| Error | 18 | 5.6 |  |
| *D. menziesii* |  |  |  |
| Intercept | 1 | 243555.3 | 21565.9*** |
| Depth | 2 | 688.7 | 61.0*** |
| UV Treatment | 2 | 218.1 | 19.3*** |
| Depth x UV Treatment | 4 | 96.8 | 8.6*** |
| Error | 18 | 11.3 |  |
| *D. anceps* |  |  |  |
| Intercept | 1 | 93904.5 | 11291.8*** |
| Depth | 2 | 145.4 | 17.5*** |
| UV Treatment | 2 | 281.3 | 33.8*** |
| Depth x UV Treatment | 4 | 417.2 | 50.2*** |
| Error | 18 | 8.3 |  |
| *H. grandifolius* |  |  |  |
| Intercept | 1 | 355067.6 | 51011.4*** |
| Depth | 2 | 425.4 | 61.1*** |
| UV Treatment | 2 | 20.3 | 2.9 ns |
| Depth x UV Treatment | 4 | 226.3 | 32.5*** |
| Error | 18 | 7.0 |  |
